# The development of a low-cost photometric-stereo-based material scanner

**DOI:** 10.1016/j.ohx.2025.e00740

**Published:** 2026-01-02

**Authors:** Lunan Wu, Federico Morosi, Giandomenico Caruso

**Affiliations:** Department of Mechanical Engineering, Politecnico di Milano, Via La Masa 1, 20156 Milan, MI, Italy

**Keywords:** Material digitisation, Photometric stereo, Custom scanner, PBR textures

## Abstract

Capturing accurate texture maps from physical materials remains a challenge in digital prototyping and projection-based spatial augmented reality (P-SAR). This paper presents an open-source material scanning system based on photometric stereo, designed for affordability, simplicity, and efficient operation. The system combines a consumer-grade digital camera, multifaceted reflector (MR16) LED lighting, and Arduino-controlled automation to acquire material data up to A4 size within 15 s. Accurate colour reproduction is achieved through a hybrid calibration workflow that integrates camera profiling with a 3D lookup table. The resulting images are processed in a streamlined Substance 3D Designer pipeline to generate albedo and normal maps compatible with physically based rendering (PBR). To evaluate performance under realistic conditions, two fabric samples were scanned and qualitatively compared with professionally digitised references. Albedo maps were assessed based on dominant colour accuracy using CIEDE2000 (ΔE_00_), while normal maps were evaluated through visual rendering comparisons and directional distribution analysis. Scanning and processing times were also measured to verify workflow efficiency. Results demonstrate that the proposed system produces perceptually consistent textures suitable for real-time rendering applications while offering a low-cost and customisable solution for material digitisation.

## Graphical abstract

**Figure:**
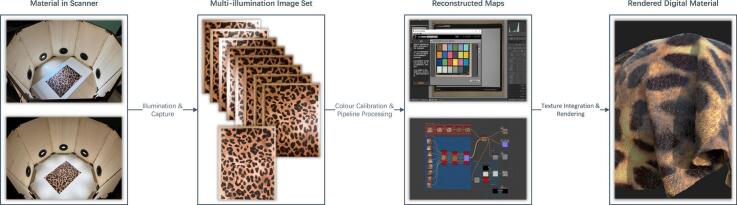

Specifications table

**Table:** 

Hardware name	Material Scanner
Subject area	• Engineering and materials science • Educational tools and open-source alternatives to existing infrastructure
Hardware type	• Imaging tools • Mechanical engineering and materials science
Closest commercial analog	X-Rite TAC7 Material Scanner
Open source license	Creative Commons Attribution-NonCommercial-ShareAlike 4.0 International
Cost of hardware	About 550 Euros plus cost of camera
Source file repository	https://doi.org/10.17632/8mprk8br23.4

## 1 Hardware in context

Physically Based Rendering (PBR) workflows have revolutionised the creation of realistic and high-quality materials, maintaining their fidelity across diverse lighting environments [1]. Unlike traditional “ad-hoc” models that require significant manual adjustments, PBR has become the standard in material creation [2]. While general-purpose PBR material generation has become more accessible, scanning specific physical materials to produce accurate digital twins remains a significant challenge for content creators [3]. These digital twins facilitate rapid iterations and optimisation in product design, enabling more efficient prototyping and creative workflows [4].

Material scanning is fundamentally an image-based 3D reconstruction process, with photogrammetry and photometric stereo being the most widely used techniques in this context [5]. Photogrammetry excels at generating geometrically accurate and dense 3D models of objects, while photometric stereo specialises in recovering detailed surface topography, even for texture-less or reflective surfaces [6], [7]. For applications focused on reproducing fine surface textures rather than overall geometry, photometric stereo offers a clear advantage [8]. Recent advancements have explored combining photometric stereo with other techniques to address its limitations, particularly low-frequency surface distortions. For instance, Nehab et al. combined 3D reconstruction data from range scanners with photometric normals, resulting in improved accuracy and detailed surface representation [9]. Similarly, Hernandez et al. employed multi-view geometric constraints derived from shape-from-silhouette (SFS) to reduce surface distortion, though this method is limited to specific parametric Bidirectional Reflectance Distribution Functions (BRDF) models [10]. Other studies have integrated photometric stereo with red–green–blue and depth (RGB-D) sensors, leveraging the detailed surface normals from photometric stereo while using RGB-D data to enhance low-frequency information [11]. These approaches, while promising, often require additional hardware or specific modelling assumptions, making them less practical for broader applications. Consequently, most commercially available material scanners continue to rely on photometric stereo for their effectiveness [12].

Photometric stereo is an effective technique for retrieving surface normals by analysing images captured under various lighting conditions [13]. Material scanners utilising photometric stereo can be broadly divided into professional-grade commercial devices and custom-designed rigs [14]. Professional scanners feature integrated designs, high precision, and factory-calibrated systems, ensuring consistent, high-resolution outputs with minimal user intervention. However, their high cost and proprietary software limit accessibility. On the other hand, custom-designed rigs, constructed using off-the-shelf components, offer cost-effective and customizable alternatives. While requiring more technical expertise for assembly and calibration, custom setups provide flexibility for specific scanning needs and are well-suited to tasks such as exploratory research or product prototyping.

X-Rite offers the TAC7 material scanner, which is a key component of its Total Appearance Capture (TAC) ecosystem [15]. The TAC7 employs 30 calibrated LED light sources and four high-resolution cameras to generate detailed PBR maps, including texture, gloss, and transparency. Supporting samples up to A4 size and 30 mm thickness, it achieves high accuracy in capturing complex surface characteristics. Scanning times range from 15 to 60 min, depending on material complexity. However, its advanced capabilities come at a significant cost of approximately €150,000, limiting its use to specialised industrial and research applications. The TAC ecosystem also includes Pantora software for managing and processing captured material data.

The HP Z Captis is another high-performance material digitisation device, featuring an advanced polarised and photometric vision system capable of capturing textures at resolutions up to 8 K [16]. Powered by the NVIDIA Jetson AGX Xavier module, it supports real-time material analysis with 32 tera operations per second (TOPS) of AI performance. The device offers two operation modes: Studio Mode, which supports samples up to 30 cm × 30 cm, including backlit translucency capture, and Explorer Mode for handling larger, flexible samples. Integrated with Adobe Substance 3D Sampler, the HP Z Captis enables seamless workflows for creating digital materials. Priced at $19,999, it is designed to meet the demands of professional users who require advanced functionality.

There are also other systems developed by companies specialising in 3D scanning technologies, providing additional options with varying capabilities and price points. Among these, the xTex A4 by Vizoo offers high-resolution texture capture with a maximum scanning area of 28 x 20 cm, utilising Nikon Z7 cameras for resolutions ranging from 630 to 950 dots per inch (DPI), depending on the lens configuration [17]. Similarly, the NunoX Premium Scanner utilises a Canon EOS R5 camera, combined with an automated lighting system, to achieve a 700 DPI resolution for material samples measuring up to 30 x 20 cm [18]. Its compact design, with dimensions of 60 x 60 x 50 cm, facilitates flexible deployment, with each scan requiring approximately 30 s. In contrast, the DMIx SamplR leverages an iPhone for capturing and a simplified one-button operation to scan flat material samples at 330 pixels per inch (PPI), making it suitable for less demanding applications [19]. Each of these devices is supported by proprietary software solutions tailored to streamline material digitisation workflows, offering tools for post-processing and integration into digital pipelines.

Several custom-engineered projects have explored innovative approaches to material scanning. The Allegorithmic team introduced a smartphone-based material scanning setup, which is seamlessly integrated into Adobe Substance 3D workflows, providing an accessible and streamlined solution [20]. Ubisoft Reflections' engineer, Grzegorz Baran, proposed an optimised tripod-based configuration, where the careful selection and arrangement of light sources significantly improved illumination quality [21]. Niklas Hauber designed a fully automated material scanner and developed an accompanying 3D reconstruction algorithm [22]. The VFX Grace team showcased the application of cross-polarisation techniques to effectively reduce glare in material scanning, enabling the capture of cleaner texture details [23].

In this paper, we introduce a material scanner leveraging photometric stereo principles to digitise physical materials into high-quality PBR textures. Key features include: 1). support for material scanning up to A4 size, comparable to commercial-grade devices; 2). a modular design allowing customisation and modification based on specific needs; 3). cost-effectiveness and ease of assembly to ensure broader accessibility; 4). an integrated software workflow with robust calibration methods; 5). user-friendly operation suitable for non-experts; 6). high accuracy in critical aspects, such as albedo colour fidelity and normal map reconstruction. By achieving a balance between performance, flexibility, and accessibility, the proposed scanner offers a practical and scalable solution for diverse material scanning applications, from prototyping to augmented reality.

## 2 Hardware description

The proposed material scanner utilises photometric stereo technology to generate high-quality PBR textures, primarily focusing on the albedo and normal maps. The system is designed with an automated mechanism for capturing raw images, while its post-processing workflow offers flexibility to adapt to various user requirements. The scanner comprises a modular mechanical framework and electronic components. In contrast to commercial proprietary systems, this modular design enables customisation of key components, such as cameras, lighting arrays, and even the scanner dimensions, to suit various applications. This adaptability, combined with low cost, makes the scanner an accessible and versatile tool for researchers and designers.

### 2.1 Mechanical framework

**Fig. 1:**
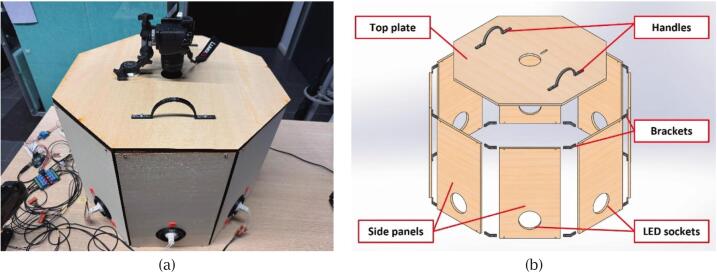
Overview of the scanner framework: (a) assembled physical prototype; (b) exploded view showing structural components.

The mechanical framework of the proposed material scanner is designed for cost efficiency and ease of assembly, leveraging laser-cut plywood and 3D-printed parts made of polyethylene terephthalate glycol (PETG). This choice of materials not only minimises manufacturing costs but also ensures rapid assembly and disassembly, making it highly accessible for researchers and designers. The framework consists of an octagonal top plate and eight side panels arranged as shown in Fig. 1.

The upper section comprises the octagonal top plate and an integrated camera mount, which together form the scanner's lid. The consumer-grade digital camera used for scanning is mounted using a standard tripod head with a 1/4–20 Unified National Coarse (UNC) thread, ensuring compatibility with widely available mounting systems. The top plate features an adjustable slot, enabling precise alignment of the camera lens with the centre of the scanned material. Once aligned, the camera bracket is secured in place using another 1/4–20 UNC thread. Users without access to a tripod may alternatively adopt an “L”-shaped bracket, which is provided as an optional design file in Section 3 (Design files summary). Additionally, the handle integrated into the top plate facilitates material replacement without disturbing the camera setup. This design ensures seamless operation while maintaining alignment accuracy, a significant advantage over rigid commercial systems that lack such flexibility.

The lower section comprises eight side panels, which are pre-cut to accommodate Light Emitting Diode (LED) lighting units. The hole diameter is slightly larger than the maximum diameter of the selected LED holder, allowing the holder to be securely inserted while ensuring a snug fit. The side panels are interconnected using upper and lower brackets, ensuring structural stability and preventing light leakage. The scanner uses MR16 LED lights, chosen for their high colour rendering index (CRI > 95) and correlated colour temperature (CCT) of 6000 K, which accurately reproduce the scanned material's colour [24], [25]. To simulate ideal parallel light sources, LEDs with narrow beam angles are utilised. Each LED is mounted in an adjustable holder, fixed with springs to allow fine-tuning of the light’s angle. These holders are inserted into pre-cut slots on the side panels, with the LED’s incidence angle ranging from 15° to 60° to achieve the desired shadow length for photometric stereo reconstruction [26]. For this study, the default angle is set to 20°, optimised for materials similar in size to A4 sheets. The side panels’ hole positions are also customisable, accommodating materials of varying dimensions and thicknesses, offering a level of flexibility not typically available in proprietary systems.

When the side panels are covered with the top plate, the resulting enclosed structure effectively blocks ambient light, creating a controlled and reproducible scanning environment. Moreover, an optional base provides additional stability when using thinner wooden panels, further enhancing cost-efficiency without compromising structural integrity.

### 2.2 Automation system

**Fig. 2:**
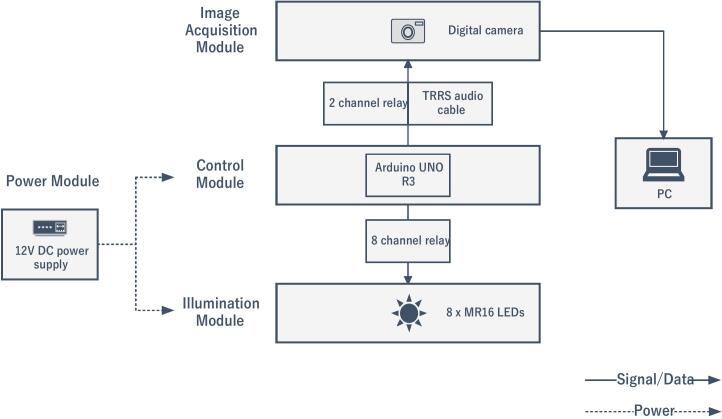
Schematic of the scanner’s automation system, including four modules.

The automation system of the proposed material scanner is designed to coordinate the functioning of the camera and LEDs. As shown in Fig. 2, the system consists of four main modules: 1). image acquisition module; 2). control module; 3). illumination module, and 4). power module.

The image acquisition module utilises a consumer-grade digital camera, specifically the Panasonic Lumix DMC-G80 in our work, which is equipped with a 64 GB SD card. The camera operates in manual mode with preset exposure parameters (ISO, aperture, and shutter speed) to ensure consistent image brightness and quality. With a maximum resolution of 4592x3448 pixels, the camera captures high-quality images essential for precise material reconstruction. The choice of exposure parameters significantly impacts reconstruction accuracy, which will be discussed further in the Operation instructions section (System setup).

The control module serves as the core of the automation system, built around an Arduino UNO R3 microcontroller. It interfaces with two relay modules to manage the illumination and camera functions. The 8-Channel Relay Module receives control signals from the Arduino to operate the eight MR16 LED lights in the illumination module, with each relay acting as an electrical switch to enable individual activation of the LEDs. Simultaneously, the 2-Channel Relay Module facilitates automatic camera shutter control by connecting the Arduino to the camera via a 3.5 mm tip–ring–ring–sleeve (TRRS) audio cable. Several resistors are incorporated into the circuit for signal modulation, with the specific resistor values varying depending on the camera model. The control logic is implemented in the C language using the Arduino IDE, enabling the program to sequentially activate the LED lights and trigger the camera shutter in a synchronised manner. This setup eliminates the need for manual operation, effectively reducing potential vibrations that could compromise image quality.

The illumination module comprises eight MR16 LED lights (equivalent to 50 W each) mounted inside the scanner’s side panels and equipped with 12 V direct current (DC) sockets. During each scanning stage, the lights are sequentially activated by the control module, forming the optical foundation for photometric stereo reconstruction. The LED selection criteria have been previously discussed and will not be reiterated here.

The power module is based on a 12 V DC switching power supply, which simultaneously powers the Arduino microcontroller and the LED lights. This shared power source simplifies the system design and ensures stable operation throughout the scanning process.

### 2.3 Post-processing workflow

**Fig. 3:**
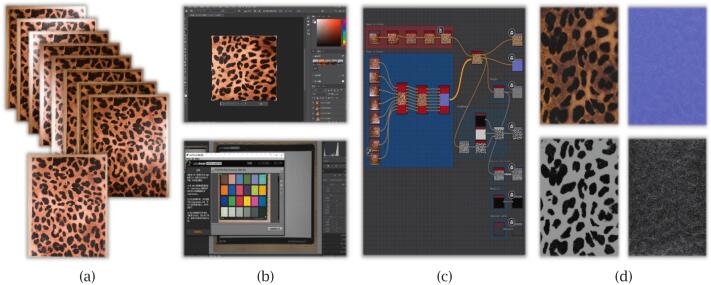
Post-processing workflow for generating PBR textures from raw scanned images: (a) input images captured by camera; (b) image pre-processing and colour calibration; (c) photometric stereo reconstruction using node-based graph; (d) output maps including albedo, normal, and others.

The post-processing workflow is designed to convert raw images captured by the scanner into high-quality PBR textures, focusing on albedo and normal maps, as shown in Fig. 3.

The process begins with colour calibration, combining both hardware and software approaches. A custom white balance is set using a neutral reference target, and a camera profile is created using a standard colour chart. Further pixel-level calibration is performed using a three-dimensional lookup table (LUT) generated from a wide set of colour samples to refine accuracy across the CIELAB colour space. After calibration, the images are manually aligned and cropped in photo editing software to ensure consistency across the image set. The valid region of interest is extracted, and all images are resized to a standard resolution of 4096 × 4096 pixels.

Texture generation is carried out in Adobe Substance 3D Designer using a custom, reusable pipeline developed within the software. The workflow consists of four main steps: image cropping, colour equalisation, photometric-stereo reconstruction, and texture tiling. Since the scanner operates with full automation, the captured images are already well aligned, eliminating the need for additional registration. Each scan is processed from nine images—one captured under full illumination for albedo reconstruction and eight directionally lit images for normal map generation. The resulting albedo and normal maps are produced at 4096 × 4096 resolution following the PBR-Metallic workflow. Additional maps, such as roughness or height, can also be generated when required. Final textures are exported in industry-standard formats (e.g., PNG and TIFF), ensuring compatibility with major rendering engines.

The choice of Adobe Substance 3D Designer was motivated by its intuitive node-based structure, which simplifies workflow design and modification [27]. The software, with an average subscription fee of €24 per month in the European region, includes built-in nodes for material scanning and photometric-stereo reconstruction, minimising the need for custom coding. Moreover, it offers excellent compatibility with widely used rendering engines such as Unity and Unreal, featuring dedicated export plugins that ensure seamless integration of the generated PBR textures.

As open-source alternatives, users may adopt the Python-based photometric-stereo implementation developed by visiont3lab, available at https://github.com/visiont3lab/photometric_stereo [28]. The reconstructed texture maps can also be further refined, combined, or exported using other open-source software such as Blender and GIMP [29], [30].

## 3 Design files summary

All design files are openly available on Mendeley Data at https://doi.org/10.17632/8mprk8br23.4 (Table 1, Table 2).

**Table 1:** List of design and software files associated with the proposed material scanning system.

Design file name	File type	Open-source license
top_plate.dwg	AutoCAD drawing file	Creative Commons Attribution-NonCommercial-ShareAlike 4.0 International
side_panel.dwg	AutoCAD drawing file	Creative Commons Attribution-NonCommercial-ShareAlike 4.0 International
base.dwg	AutoCAD drawing file	Creative Commons Attribution-NonCommercial-ShareAlike 4.0 International
camera_mount_a.dwg	AutoCAD drawing file	Creative Commons Attribution-NonCommercial-ShareAlike 4.0 International
camera_mount_b.dwg	AutoCAD drawing file	Creative Commons Attribution-NonCommercial-ShareAlike 4.0 International
side_bracket.sldprt	SolidWorks part file	Creative Commons Attribution-NonCommercial-ShareAlike 4.0 International
handle.sldprt	SolidWorks part file	Creative Commons Attribution-NonCommercial-ShareAlike 4.0 International
control_circuit_diagram.pdf	PDF file	Creative Commons Attribution-NonCommercial-ShareAlike 4.0 International
control_circuit_components.pdf	PDF file	Creative Commons Attribution-NonCommercial-ShareAlike 4.0 International
calibration_program.ino	Arduino sketch file	Creative Commons Attribution-NonCommercial-ShareAlike 4.0 International
scan_program.ino	Arduino sketch file	Creative Commons Attribution-NonCommercial-ShareAlike 4.0 International
colour_selection.m	MATLAB script file	Creative Commons Attribution-NonCommercial-ShareAlike 4.0 International
three_d_lut.m	MATLAB script file	Creative Commons Attribution-NonCommercial-ShareAlike 4.0 International
colour_calibration.m	MATLAB script file	Creative Commons Attribution-NonCommercial-ShareAlike 4.0 International
post_process_pipeline.sbs	Substance 3D source file	Creative Commons Attribution-NonCommercial-ShareAlike 4.0 International
post_process_documentation.pdf	PDF file	Creative Commons Attribution-NonCommercial-ShareAlike 4.0 International

**Table 2:** List of design and software files associated with the proposed material scanning system.

Design file name	Description
top_plate.dwg	This file contains the laser-cut design for the scanner's top plate, which serves as the structural lid and supports the camera mount and handle.
side_panel.dwg	This file contains the laser-cut design for the scanner's side panels, including pre-cut holes for mounting LED lights.
base.dwg	This file contains the laser-cut design for the scanner's optional base, which enhances stability.
camera_mount_a.dwg	This file contains part of the laser-cut design for the adjustable camera mount bracket, ensuring precise alignment of the camera lens.
camera_mount_b.dwg	This file contains part of the laser-cut design for the adjustable camera mount bracket, ensuring precise alignment of the camera lens.
side_bracket.sldprt	This file contains the 3D printable design for the side brackets, used to connect the side panels securely.
handle.sldprt	This file contains the 3D printable design for the handle attached to the top plate, allowing easy removal of the lid.
control_circuit_diagram.pdf	This file contains the schematic diagram of the control system, showing connections between the Arduino, relay modules, LEDs, and camera.
control_circuit_components.pdf	This file contains a detailed list of electronic components used in the control circuit.
calibration_program.ino	This file contains the Arduino program for the calibration process, including lighting and camera triggering.
scan_program.ino	This file contains the Arduino program for automating the scanning process, including sequential LED activation and camera control.
colour_selection.m	This file contains a MATLAB script used to manually select colour patches from images of the colour samples.
three_d_lut.m	This file contains a MATLAB script that constructs a 3D LUT by comparing the recorded values with predefined reference values.
colour_calibration.m	This file contains a MATLAB script that applies the previously generated 3D LUT to the fully illuminated image for precise colour correction.
post_process_pipeline.sbs	This file contains the custom node-based template for Adobe Substance 3D Designer, designed for texture maps reconstruction.
post_process_documentation.pdf	This file contains detailed information on the Substance 3D Designer pipeline.

## 4 Bill of materials summary

• P1 controls the sequential activation of MR16 LED bulbs and the synchronised triggering of the camera shutter. (Table 3)

**Table 3:** Bill of materials and cost overview for the proposed material scanning system.

Designator	Component	Number	Cost per unit −currency	Total cost − currency	Source of materials	Material type
P1	Arduino UNO Rev 3	1	29,80 EUR	29,80 EUR	Amazon	Other
P2	USB-A to USB-B Cable	1	3,51 EUR	3,51 EUR	AliExpress	Other
P3	5 V 8-Channel Relay Module	1	6,49 EUR	6,49 EUR	AliExpress	Other
P4	5 V 2-Channel Relay Module	1	1,72 EUR	1,72 EUR	AliExpress	Other
P5	Jumper Wire Kit 120-pcs	1	9,98 EUR	9,98 EUR	Amazon	Other
P6	Solderless Breadboard	2	1,58 EUR	3,16 EUR	AliExpress	Other
P7	12 V 8.5A Switching Power Supply	1	28,59 EUR	28,59 EUR	AliExpress	Other
P8	Silicone Electrical Wire 20-meter	1	13,85 EUR	13,85 EUR	AliExpress	Other
P9	5-Conductor Connection Terminal 10-pcs	1	4,97 EUR	4,97 EUR	AliExpress	Other
P10	2-Conductor Connection Terminal 10-pcs	2	3,46 EUR	6,92 EUR	AliExpress	Other
P11	GU5.3 MR16 LED Spotlight 6 pcs	2	18,74 EUR	37,48 EUR	Amazon	Other
P12	MR16 LED DC Socket & Adjustable Holder	8	4,82 EUR	38,56 EUR	AliExpress	Other
P13	2.5 mm to 3.5 mm TRRS Audio Cable	1	3,02 EUR	3,02 EUR	AliExpress	Other
P14	Carbon Film Resistor Kit	1	14,61 EUR	14,61 EUR	AliExpress	Other
P15	Laser Cutting Service	1	256,00 EUR	256,00 EUR	Protosign Srl	Composite
P16	3D Printing Service	1	51,65 EUR	51,65 EUR	Xometry GmbH	Polymer
P17	M3x20 mm Screw Bolt Set 30-pcs	2	2,15 EUR	4,30 EUR	Bricocenter	Metal
P18	M4x20 mm Screw Bolt Set 20-pcs	1	1,95 EUR	1,95 EUR	Bricocenter	Metal
P19	Circular Polarising Filter	1	22,99 EUR	22,99 EUR	Amazon	Other
P20	Linear Polarising Film 2-pcs	1	21,99 EUR	21,99 EUR	Amazon	Other
• P2 connects the Arduino to a computer for power supply and programming.
• P3 switches the MR16 LED bulbs during the scanning process, enabling automated illumination control.
• P4, P6, P7, P8, P9, and P10 set up the circuit for automatically controlling the MR16 LED switches and providing DC power to them during the scanning process via the Arduino.
• P5, P6, P13, and P14 set up the circuit for automatically controlling the camera shutter during the scanning process via the Arduino.
• P12 secures the MR16 LEDs and allows the adjustment of lighting angles.
• P15 covers the cost of laser cutting the structural components, including the top plate, side panels, camera mount bracket, and base.
• P16 covers the cost of 3D printing the components, such as the side brackets and the handle.
• P17 secures the adjacent side panels and the side brackets.
• P18 secures the handle onto the top plate.
• P19 and P20 are used for cross-polarisation during scanning to reduce glare.

### 4.1 Software used

• Dassault SolidWorks: Used for 3D modelling of the scanner’s structural components.
• Autodesk AutoCAD: Used for creating engineering drawings for laser cutting and hardware assembly.
• Fritzing: Used for designing the control circuit diagram.
• Arduino IDE: Used for writing and uploading automation scripts to the Arduino.
• X-Rite ColorChecker Camera Calibration: Used to generate a DNG Camera Profile (DCP) for standardising the camera’s colour response.
• Adobe Lightroom Classic: Used for editing raw images, including white balance correction and initial colour calibration with DCP profiles.
• MathWorks MATLAB: Used to develop a custom 3D LUT script for advanced colour calibration of scanned images.
• Adobe Photoshop: Used for aligning and cropping calibrated images and resizing them to a standard resolution before texture reconstruction.
• Adobe Substance 3D Designer: Used for reconstructing PBR textures with a node-based workflow.

### 4.2 Tools used

• Screwdrivers
• Wire cutter and stripper
• Crimping tool
• Multimeter
• Paper tape
• Hot-melt adhesive
• X-Rite ColorChecker White Balance & Classic
• RAL D2 Color Fan

## 5 Build instructions

The construction of the material scanner involves three main stages: fabricating the components, assembling the mechanical framework, and integrating the electronics system. This process is designed to be reproducible, with all necessary design files and materials listed above.

### 5.1 Fabrication of components

The fabrication process involves producing the scanner’s structural and functional components, including laser-cut parts, 3D-printed parts, and polarisation elements.

• Laser-cut components (Table 4)

**Table 4:** Laser-cut components and fabrication details.

Component	Design file	Process	Material	Material thickness
Top Plate	top_plate.dwg	CO_2_ laser cutting	Plywood	10 mm
Side Panel	side_panel.dwg	CO_2_ laser cutting	Plywood	10 mm
Camera Mount Bracket	camera_mount_a.dwgcamera_mount_b.dwg	CO_2_ laser cutting	Plywood	10 mm
Base	base.dwg	CO_2_ laser cutting	Plywood	10 mm

Adjust the relevant design dimensions according to the selected LED bulb size and camera lens diameter to ensure proper fit during assembly.

Import the provided.dwg files into the laser cutter software or export them to.dxf if the machine is not compatible with.dwg. Configure the cutting parameters based on the 10 mm plywood thickness, then position the sheets on the laser cutter bed, ensuring proper alignment to minimise material wastage. Once completed, label each component to facilitate identification during the assembly process.

• 3D-printed components (Table 5)

**Table 5:** 3D-printed components and fabrication details.

Component	Design file	Process	Material	Material infill
Side Bracket	side_bracket.sldprt	FDM 3D printing	PETG	50–80 %
Handle	handle.sldprt	FDM 3D printing	PETG	20–50 %

PETG is used for the side brackets and handle due to its strength and flexibility, which prevent cracking under stress during assembly and use. Additionally, PETG is recyclable and emits minimal fumes during printing. To fabricate these components, open the provided SolidWorks part (.sldprt) files and export them as STL files. Import the STL files into a slicing software and configure the printing parameters, including the infill percentage as indicated in the table above and a layer thickness of 200 µm to balance print quality and speed. Once completed, inspect each part for dimensional accuracy and remove any support structures or excess material.

• Polarisation components (Table 6)

**Table 6:** Polarisation components used in the material scanning system.

Component	Description
Circular Polarising Filter	A ready-to-use round filter matching the camera lens diameter
Linear Polarising Film	Rectangular sheets requiring cutting before use

The polarisation components are employed to reduce specular glare and enable the acquisition of clear albedo maps of materials [31]. If the linear polarising film has an adhesive side, it can be cut into round pieces matching the LED size and directly affixed to the front surface of each LED. Otherwise, the film can be cut into similarly sized discs and inserted between the LED bulb and its holder. In both cases, a small notch should be added during cutting to indicate the polarisation direction, which corresponds to the long edge of the original rectangular film sheet.

**Fig. 4:**
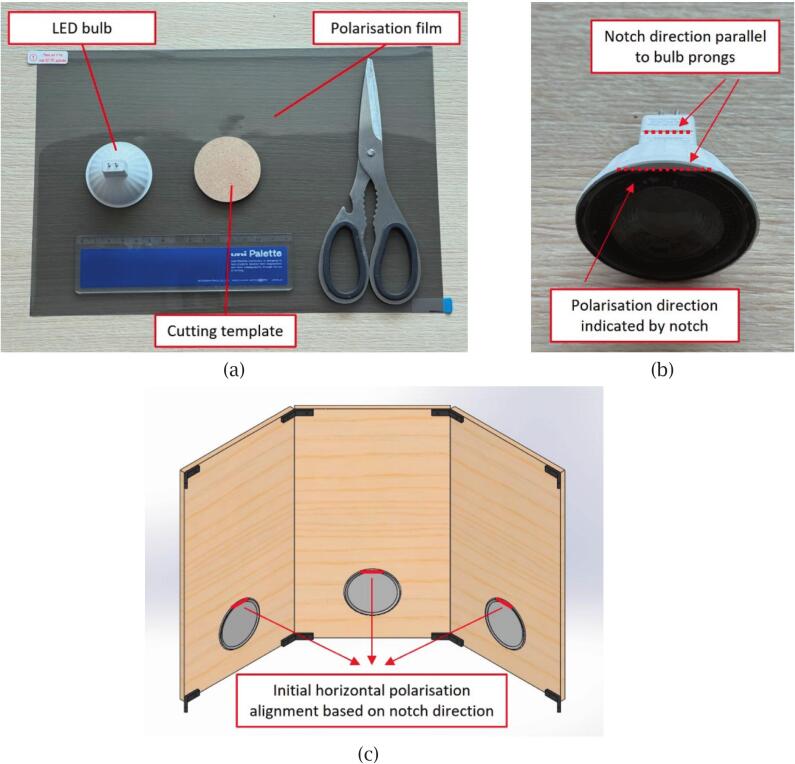
Initial polarisation setup for LEDs: (a) Tools and materials for cutting circular polarising film, including a cutting template matching the LED diameter; (b) LED bulb with mounted polarising film — the red notch indicates the polarisation direction, which is aligned parallel to the rear prongs for consistent orientation; (c) CAD illustration of the assembled scanner, showing the initial horizontal alignment of polarisation direction across all LEDs based on the notch orientation.

When mounting the film, the notch should be aligned parallel to the rear insertion prongs of the LED bulb. This ensures that all polarising films are installed with a consistent horizontal orientation. This initial alignment does not establish cross-polarisation but rather serves to simplify its subsequent calibration. The procedure is illustrated in Fig. 4.

The actual cross-polarisation configuration, which depends on the final orientation of the LEDs within the scanner structure, is described in the following section, “Assembly of the mechanical framework”, where the LED units are integrated with the side panels and calibrated accordingly.

### 5.2 Assembly of the mechanical framework

The mechanical framework of the scanner is assembled in two sections: the lower section, which forms the base and main support structure, and the upper section, which houses the camera mount and removable top plate.

• Upper section

The upper section consists of the camera mount bracket, the top plate, and the handle, forming the support structure for the camera and providing easy access to the scanner’s interior.

**Fig. 5:**
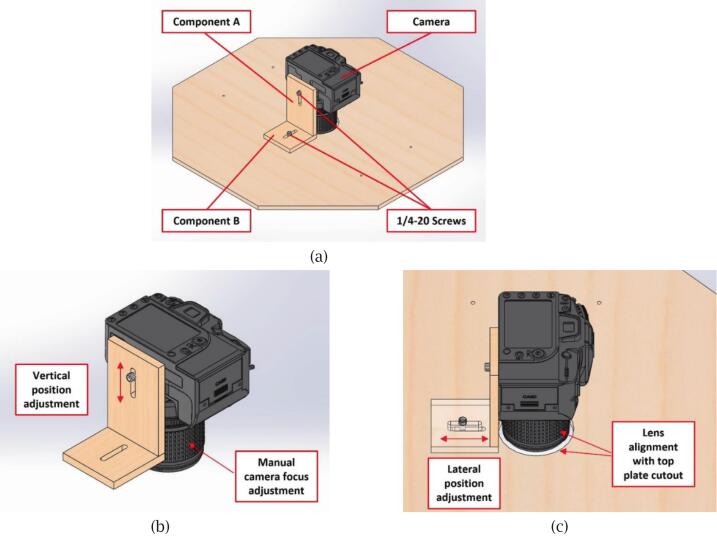
Camera mount bracket: (a) Assembled L-shaped bracket composed of components A and B; (b) Vertical adjustment enabled by the slot in component A; (c) Lateral adjustment via component B to align the lens precisely with the circular cutout on the top plate.

The camera mount bracket is assembled by bonding components A and B together using hot-melt adhesive, forming an L-shaped structure, as shown in Fig. 5. Component A has an elongated slot and uses a standard 1/4–20 UNC thread to mount the camera facing downward. The camera's position can be adjusted vertically along this slot, allowing it to capture different material sizes placed at the base of the scanner, in conjunction with the camera’s focus adjustment. Component B also features an elongated slot with a 1/4–20 UNC thread, which secures the bracket to the top plate. This configuration enables horizontal camera adjustment to ensure that the lens is aligned with the circular cutout at the centre of the top plate.

**Fig. 6:**
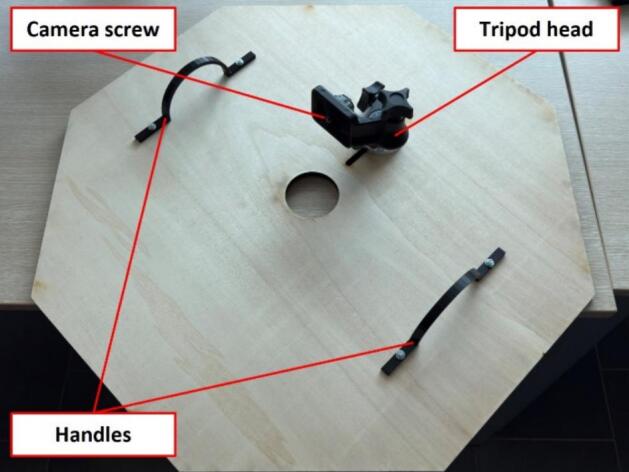
Alternative camera mounting method using a tripod head.

If a ready-made tripod head is available, it can replace the L-shaped camera mount bracket, eliminating the need for fabrication and assembly, as shown in Fig. 6. The tripod head can be directly attached to the top plate, providing an adjustable and stable mounting solution for the camera.

Finally, the handle is secured to the top plate using M4x20 screws, fastened through the pre-drilled holes on both sides, as illustrated in Fig. 6. This handle allows for easy removal of the top plate without disturbing the camera setup, making it convenient to replace scanning materials. Once the camera mount bracket and handle are installed, the upper section assembly is complete and ready for integration with the lower section.

• Lower section

The lower section comprises the eight side panels, sixteen side brackets, and LED assemblies, which form the main framework of the scanner.

**Fig. 7:**
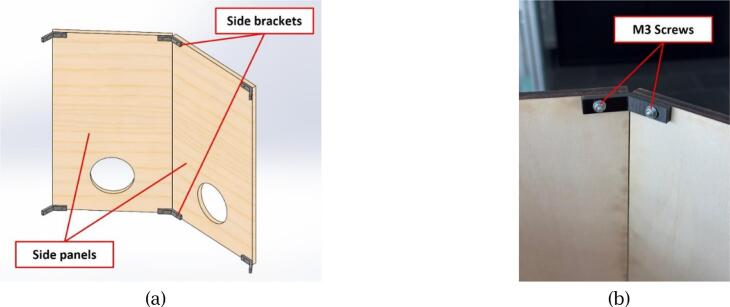
Assembly detail of side panels: (a) Two adjacent side panels connected using top and bottom side brackets; (b) Close-up of screws fixing the bracket to the panel.

To begin, assemble the eight side panels into an octagonal frame. Each pair of adjacent panels is connected using two side brackets, one at the top and one at the bottom, and secured with M3x20 screws, as shown in Fig. 7.

If the side panels are made from thinner plywood, an optional base can be added to enhance structural stability. This base is cut from the same plywood material using excess material from the panel fabrication process. During assembly, the side panels are inserted into the base frame, providing additional support and ensuring a more rigid structure.

**Fig. 8:**
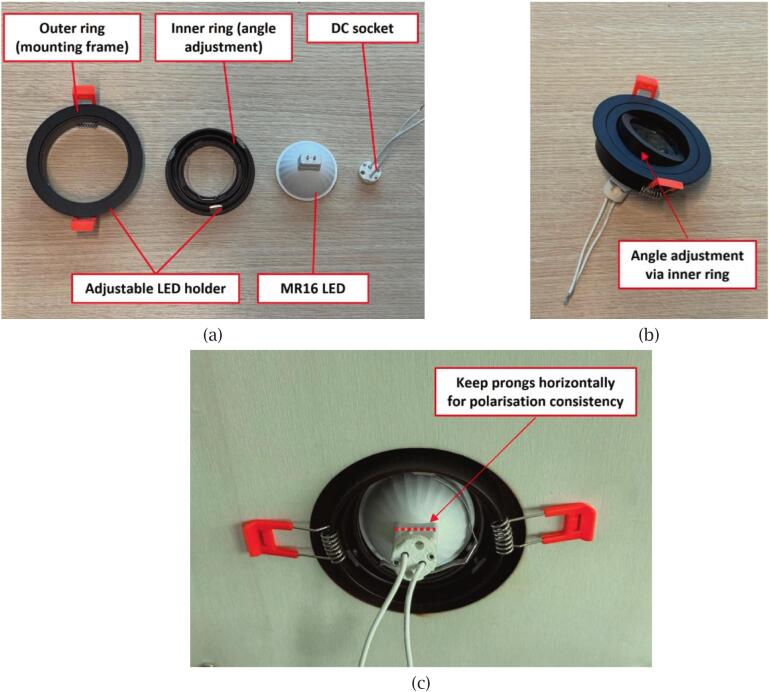
LED assembly process: (a) Components of the LED unit; (b) Assembled LED unit with adjustable angle via inner ring; (c) Installed LED unit with rear prongs aligned horizontally.

Once the frame is assembled, install the LED assemblies, each consisting of an MR16 LED bulb, a DC socket, and an adjustable LED holder, as illustrated in Fig. 8. First, insert the two pins of the LED bulb into the corresponding slots on the DC socket to establish the electrical connection. Following the setup described in the polarisation components section, place the LED bulb inside the LED holder. Adjust the holder angle as needed to achieve the desired illumination direction (typically between 15° and 60°); in this study, a 20° angle was selected.

Next, insert the assembled LED units into the pre-cut holes on each side panel. The spring-loaded sides of the LED holder will snap into place, securing the LED unit firmly within the panel, as shown in Fig. 8. Ensure that the rear insertion prongs of each LED remain horizontal to maintain the correct polarisation alignment, as described in the Polarisation components section. Repeat this process for all eight LED assemblies, completing the lower section assembly.

Once all LEDs are installed, the cross-polarisation configuration can be established. Begin by turning on all LEDs using the provided calibration programme, and activate the camera with a circular polarising filter (CPL) attached. Slowly rotate the CPL filter while observing the live camera preview until one pair of opposite LEDs appears darkest. This indicates that their polarisation direction is perpendicular to the current CPL orientation. At this point, the remaining three LED pairs should be manually adjusted by rotating the rear insertion prongs of each LED by 90 degrees. Care must be taken to maintain the original tilt angle of the LED holders during this adjustment. Once all eight LEDs show minimal visible light under the same CPL setting, the cross-polarisation setup is complete.

Finally, place the upper section of the scanner onto the lower section, ensuring proper alignment between the top plate and the side panels. Confirm that the camera mount remains centred above the scanning area. This final step completes the assembly of the mechanical framework, resulting in a stable and enclosed structure ready for material scanning.

### 5.3 Integration of electronics system

**Fig. 9:**
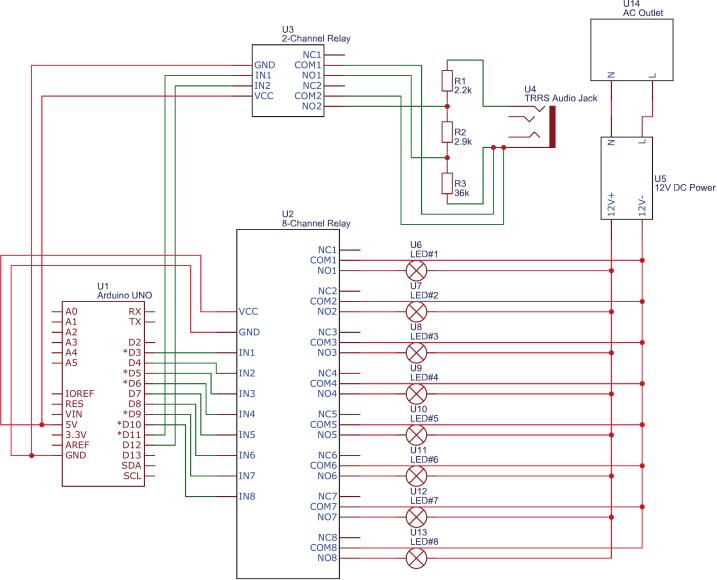
Wiring diagram for the scanner, including LED control and camera shutter triggering. Green lines indicate control signals; red lines represent power connections. (For interpretation of the references to colour in this figure legend, the reader is referred to the web version of this article.)

The electronics system of the scanner comprises two main subsystems: the LED lighting control subsystem and the camera shutter control subsystem. Fig. 9 shows the wiring diagram used to implement both subsystems. The green lines represent control signals sent from the Arduino to the relay modules, while the red lines indicate power delivery to the MR16 LED bulbs and the camera’s shutter interface. The complete wiring diagram is also provided as control_circuit_diagram.pdf in the repository.

• Lighting control circuit

The lighting system is designed to control the eight MR16 LED bulbs individually, allowing for sequential illumination during scanning. The circuit is based on an Arduino UNO (P1), which sends signals to a 5 V 8-channel relay module (P3) to switch the LEDs on and off.

To set up the circuit, first connect the Arduino’s digital output pins to the relay module’s signal input pins using jumper wires (P5). Each relay corresponds to a single LED and is triggered sequentially according to the scanning program. The MR16 LEDs (P11) are powered by a 12 V 8.5A switching power supply (P7), with power routed through the relay module to control activation. Silicone electrical wires (P8) and connection terminals (P9, P10) are used to link the LEDs and power supply.

Once all connections are made, verify the circuit functionality by uploading the scan program (scan_program.ino) to the Arduino. Even before integrating the camera control circuit, running the scan program allows verification that each LED turns on and off as expected in the correct sequence. Avoid staring directly at the high-brightness LED bulbs for extended periods, as this may cause visual discomfort or potential eye strain.

• Camera control circuit

The camera control system ensures precise synchronisation between the LED activation sequence and the camera shutter trigger. This is achieved using a 5 V 2-channel relay module (P4), which simulates a physical shutter button press via a TRRS audio cable (P13) connected to the camera’s remote trigger input.

To build the shutter control circuit, begin by cutting off the 3.5 mm end of the TRRS audio cable (P13) to expose its four internal wires, which correspond to tip (T), ring 1 (R1), ring 2 (R2), and sleeve (S). Using a multimeter, identify the wires corresponding to Ring 2 and Sleeve by probing the metal segments of the TRRS plug and testing for continuity with the exposed wires. These two wires are required to simulate the shutter mechanism.

**Fig. 10:**
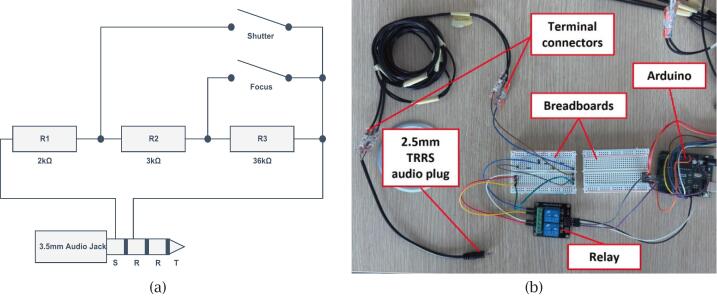
Camera shutter control circuit for Panasonic Lumix cameras: (a) Schematic diagram using resistors and a 2.5 mm TRRS jack to simulate shutter and focus signals; (b) Physical setup showing Arduino, relay, breadboard, and the TRRS wiring interface.

The schematic shown in Fig. 10(a) illustrates the circuit built on the cut (3.5 mm) end of the TRRS cable, which connects to the relay-controlled resistor network (2 kΩ, 3 kΩ, and 36 kΩ from P14). When the Arduino’s digital output pin triggers the relay, it momentarily closes the appropriate circuit, signalling the camera to capture an image.

The 2.5 mm end of the TRRS cable remains intact and is inserted into the remote input port of a Panasonic Lumix camera, as shown in Fig. 10(b). This circuit configuration is specifically designed for Lumix cameras; for other camera brands, compatible circuits can be found online.

After connecting the camera control circuit, re-run the scan program (scan_program.ino) and verify that the camera shutter triggers at the correct intervals, matching the LED activation sequence. If necessary, timing parameters in the scan program can be adjusted for optimal synchronisation.

During the wiring process of both circuits, use a solderless breadboard (P6) to facilitate connections and allow for easy adjustments before finalising the setup.

## 6 Operation instructions

The following instructions outline the proper use of the scanner, including system setup, calibration, scanning, and data processing. Ensure that all hardware is correctly assembled and connected as described in Section “5. Build instructions.”

• System setup

1) Positioning the scanner

**Fig. 11:**
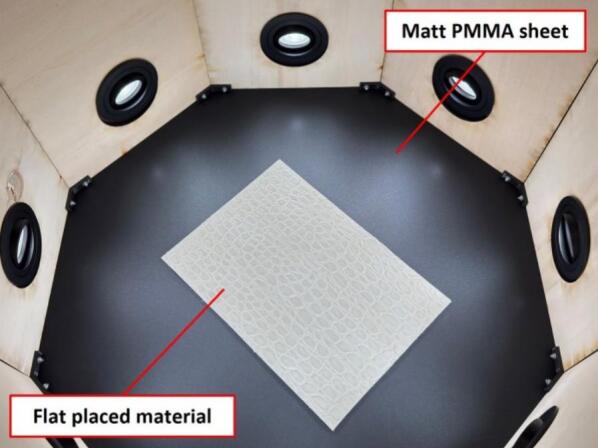
Internal view of the scanner with the material placed on a matte black background.

Place the lower portion of the scanner on a flat matte-black acrylic sheet or opaque black paperboard, which will serve as the background during the scanning process. This helps minimise reflections and enhances contrast for better texture extraction. Next, place the material to be scanned inside the lower portion of the scanner, ensuring that it lies flat on the background. An overview of this setup is illustrated in Fig. 11. The material dimensions should not exceed 300 × 300 mm to fit within the scanning area in this configuration. Once the material is positioned, carefully lower the upper portion onto the scanner, ensuring that all edges align properly with no visible gaps.

2) Mounting the camera

Attach the camera to the L-shaped camera mount or a tripod head, securing it with a 1/4–20 UNC screw. Adjust the camera's horizontal position so that the lens is centred with the circular cutout on the top plate. Adjust the vertical position as needed to ensure the entire material is visible within the camera’s field of view. Turn on the camera and switch to manual focusing (MF) mode. Observe the viewfinder and adjust both the camera mounting bracket position and the camera’s focus ring until the entire material is in sharp focus.

The camera is set to manual exposure mode (M) and maintains fixed exposure parameters throughout all scanning sessions. To balance exposure and consistency, a standard configuration is used: ISO: 100 or lower (minimising noise due to the high brightness of LED lights), aperture: f/11 or f/16 (two stops below maximum to ensure depth of field), shutter speed: 1/60″ (faster than the safe shutter value, determined based on LED brightness and focal length) [32].

Besides, set the camera to its maximum resolution and select the highest quality storage format (RAW format).

3) Configuring the Arduino

Connect the Arduino controller to the computer’s USB port using a USB-A to USB-B cable (P2). Open Arduino IDE, then navigate to Tools > Port and check if a port name appears. Try reconnecting the cable using a different USB port if no port is detected. Finally, connect the customised 3.5 mm TRRS audio cable to the camera’s remote trigger input.

• Colour calibration

1) Lighting setup for calibration

Once the Arduino connection is established, open the calibration lighting control program (calibration_program.ino) in Arduino IDE and upload the script to the Arduino controller. This program ensures that all LEDs are turned on, providing the same illumination conditions as used during the albedo scanning phase. This lighting setup must remain consistent throughout all stages of calibration.

2) Hardware-based calibration

To standardise colour reproduction, the scanner undergoes an initial calibration using X-Rite ColorChecker tools under the same lighting conditions, camera settings, and exposure parameters as the scanning workflow.

**Fig. 12:**
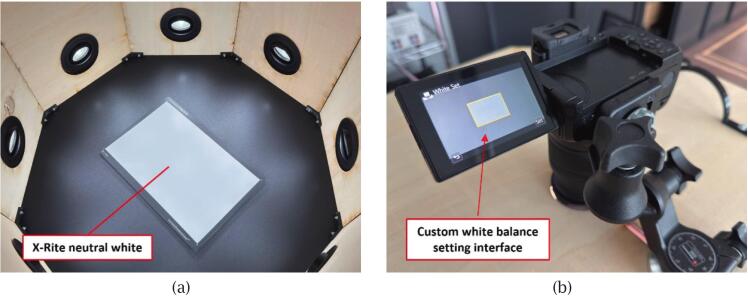
White balance calibration using the X-Rite Neutral White card: (a) Placement of the neutral target inside the scanner; (b) Camera interface used to configure custom white balance.

Place the X-Rite ColorChecker White Balance card inside the scanner and adjust the lighting to the preset scanning configuration. As shown in Fig. 12, use the camera’s custom white balance function and press the shutter button to confirm the setting. Ensure that the neutral white area of the card fully fills the central frame on the camera screen during this process to guarantee accurate calibration.

**Fig. 13:**
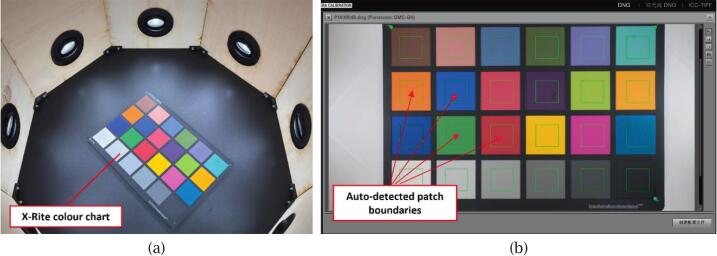
DCP profile generation using the X-Rite ColorChecker Classic chart: (a) Chart placement inside the scanner for image capture; (b) Automatic detection of the 24 colour patches in X-Rite calibration software.

Next, replace the white reference with the ColorChecker Classic chart and capture another image (Fig. 13). Convert this image to DNG format and import it into X-Rite ColorChecker Camera Calibration software to generate a DCP camera profile, which is later applied to all RAW images in Adobe Lightroom Classic. The software will automatically detect the 24 colour patches of the ColorChecker Classic chart within the image; if automatic detection fails, manual alignment of the patch grid can still be performed.

3) Software-based calibration

While the DCP camera profile provides an overall hue correction, further refinement is performed through a 3D lookup table (3D LUT) calibration [33].

**Fig. 14:**
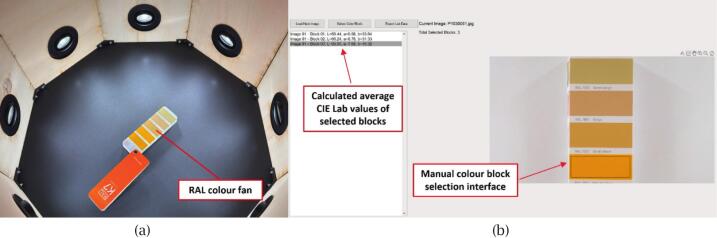
3D LUT generation using RAL colour samples: (a) Placement of RAL colour fan inside the scanner for image capture; (b) Manual selection of colour blocks and automatic calculation of average CIE Lab values in MATLAB.

First, capture a series of RAL colour samples, such as those from the RAL D2 colour fan, as illustrated in Fig. 14. Load these images into MATLAB and run the script (colour_selection.m), which allows the user to manually select colour patches from the images. The script calculates the average L, a, and b values for each sample, representing the camera’s recorded colours, and automatically stores them for further processing. After selecting all colours from captured images, run another script (three_d_lut.m), which automatically compares the recorded values with the user-provided reference L, a, and b values (imported as a.mat file and placed in the designated directory, reference data available online), and then applies trilinear interpolation to generate and store the final 3D LUT.

Once the 3D LUT is generated, keep MATLAB open, as it will be needed later to run the other script (colour_calibration.m) for colour correction using the 3D LUT after scanning.

• Scanning

1) Capturing raw images

**Fig. 15:**
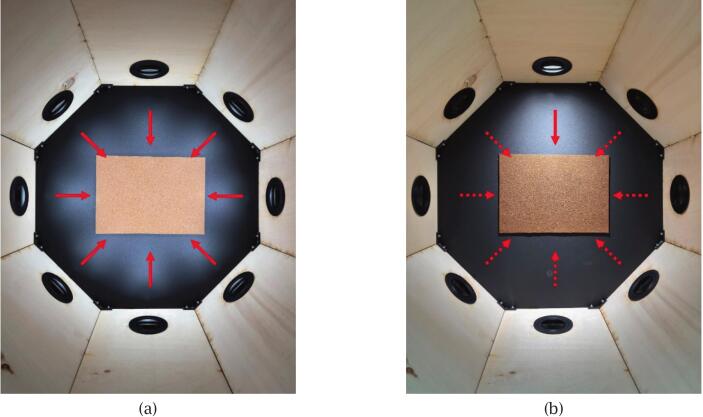
Lighting configurations during scanning: (a) Full illumination for albedo reconstruction; (b) Directional lighting for normal map reconstruction. Solid arrows indicate active LEDs; dashed arrows indicate inactive ones.

Connect the DC switching power supply to an AC outlet to power the MR16 LED system. Then, connect the Arduino board to a computer via USB and upload the scan program (scan_program.ino) using the Arduino IDE. Once the program is successfully uploaded, the scanning process will automatically initiate, during which the system will sequentially activate the MR16 LEDs while triggering the camera shutter. The scan captures one fully illuminated image for albedo reconstruction, and eight images with directional lighting for normal map reconstruction, as shown in Fig. 15.

2) Verifying scanned images

Remove the SD card from the camera and check the stored images to ensure that all lighting stages have been correctly captured. If any misalignment, exposure issues, or missing images are detected, adjust the setup and repeat the scan if necessary.

• Data processing

1) Correcting raw images

For optimal performance, a high-performance computer with a dedicated GPU is recommended. In this study, an Intel Core i7 processor, NVIDIA RTX 2060 GPU, and 32 GB RAM were used to ensure smooth processing.

Transfer the scanned images to the computer and import them into Adobe Lightroom Classic. Note that only the fully illuminated image used for albedo reconstruction requires colour calibration. Apply the previously generated DCP camera profile to standardise colour reproduction. After processing, export all the images in 16-bit TIFF format to preserve details for further reconstruction.

To further refine colour accuracy, load the fully illuminated image into MATLAB and run the script (colour_calibration.m). This script applies the 3D LUT, adjusting each pixel’s L, a, and b values to match the reference colour samples. This corrected image, along with the eight other images, is then used in the texture reconstruction process.

Next, import all nine images in Adobe Photoshop using the Load Files into Stack function, making sure to enable Auto-align Layers to compensate for any sub-pixel shifts during scanning. Then, crop the image stack into a square region that fully contains the material while minimising background content. Resize the cropped stack to 4096 × 4096 pixels using the Preserve Details 2.0 resampling algorithm to maintain texture fidelity. Finally, export each layer as a separate TIFF file for use in the reconstruction pipeline.

2) Reconstructing textures

**Fig. 16:**
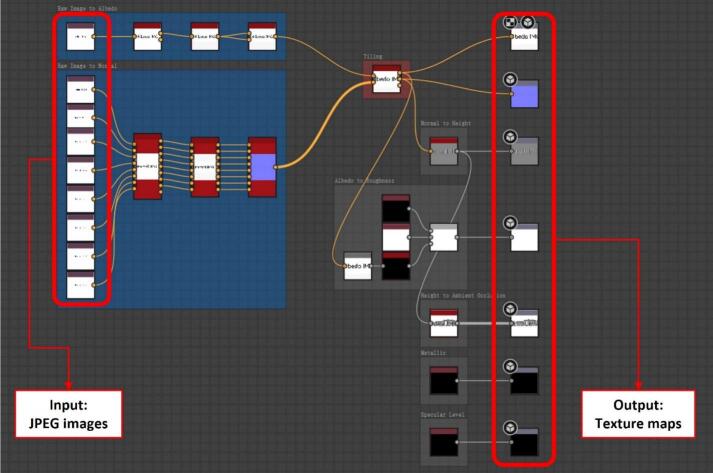
Pre-configured node graph in Adobe Substance 3D Designer for texture reconstruction.

Open Adobe Substance 3D Designer and load the provided template file (post_process_pipeline.sbs). This node-based pipeline is pre-configured for material reconstruction and is visually structured using color-coded blocks for clarity, as shown in Fig. 16. For a detailed description of nodes and their parameter settings, refer to the supplementary document (post_process_documentation.pdf) available in the repository. This document provides a high-resolution version of the workflow, explanations of the key processing nodes, and input/output examples to facilitate accurate reproduction of the reconstruction process.

Import the nine images into the pipeline, replacing the placeholder white images. Assign the one fully illuminated image to the “Image to Albedo” block and the eight images with directional lighting to the “Image to Normal” block. Carefully select the correct normal format (OpenGL or DirectX) to match the intended rendering engine. Additionally, ensure that the imported images follow the correct light direction sequence (clockwise or counterclockwise) to produce an accurate representation of the surface normal map.

Adjust key processing parameters, including cropping (Multi Crop node), colour equalisation (Multi Colour Equalizer node), and tiling adjustments (Smart Auto Tile node). When setting up the crop node, adjust the input size to match the actual pixel resolution of the input images to avoid stretching. Observe the real-time rendered material 2D/3D preview in Substance 3D Designer, fine-tuning the parameters until the generated texture meets the desired appearance. Ultimately, this node graph primarily generates two PBR textures: the albedo map and the normal map. If needed, additional texture maps such as height, roughness, and metallic maps can be generated using the corresponding blocks already included in the template pipeline.

3) Exporting texture maps

Finally, export all generated PBR texture maps, with the default resolution set to 4096x4096 in the provided template. Adobe Substance 3D Designer allows exporting texture maps in various formats, including TIFF, PNG, and PSB. When exporting, ensure that each texture map is saved in the appropriate colour space—for example, use sRGB for albedo and normal maps and linear colour space for height, roughness, and metallic maps.

These exported texture maps can be directly integrated into 3D rendering engines such as Unity, Unreal Engine, and Blender, enabling the creation of high-quality materials for real-time visualisation and physically based rendering workflows.

## 7 Validation and characterisation

One of the intended application scenarios for the proposed scanning system is the digitisation of fabric materials for use in P-SAR systems. In this context, digitised textures are projected onto white physical prototypes to simulate real materials during design reviews, allowing designers to evaluate material appearance without the need for physical samples or fabrication.

This application imposes specific constraints on the quality of the generated textures. Since P-SAR relies on real-time rendering, overly detailed or high-resolution textures may introduce computational overhead and reduce system responsiveness. Moreover, the visual fidelity of the projected output is inherently limited by the resolution and optical characteristics of the projector. Therefore, it is sufficient for textures to appear visually plausible and consistent under projection, rather than photo-realistically accurate.

To evaluate whether the proposed system meets these requirements, we conducted a material-oriented validation using two fabric samples, each measuring approximately the size of an A4 sheet (additional details related to the validation procedure and methodology can be found in [37]). Sample 1 is a warm brown synthetic leather featuring a fine, evenly distributed, pebble-like texture. Sample 2 is a woven fabric composed of alternating black and off-white vertical stripes, presenting strong directional texture and high contrast. These fabrics were also scanned using a professional material digitisation system, and the results were used as qualitative references. The validation procedure included:

1) Visual and quantitative assessment of albedo map colour accuracy, based on dominant colour tones and perceptual colour difference;
2) Visual and directional evaluation of normal maps, including rendered appearance and spherical distribution analysis of surface normals;
3) Measurement of scanning and post-processing time, to assess the operational efficiency and consistency of the overall workflow.

### 7.1 Albedo map assessment

The evaluation of albedo maps considered both visual and numerical criteria aligned with the requirements of projection-based rendering. Both fabric samples were reconstructed under a consistent, calibrated lighting setup.

• Visual Inspection

**Fig. 17:**
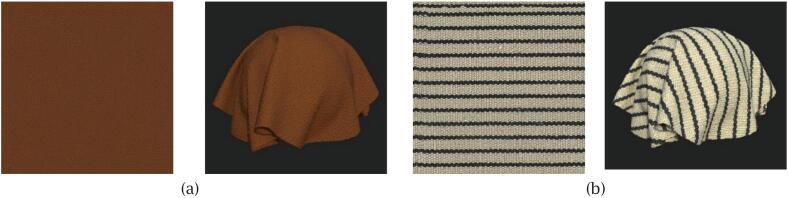
Albedo maps and rendered appearances of the two scanned fabric samples: (a) brown synthetic leather; (b) striped woven fabric.

The reconstructed albedo maps were visually inspected to assess the perceptual quality of surface appearance. As shown in Fig. 17, sample 1 is a warm brown synthetic leather with a uniformly distributed pebble-like texture. The reconstructed albedo map successfully preserved the consistent colour tone and fine surface detail, with no visible artefacts such as residual highlights or shading inconsistencies. Sample 2 is a striped woven fabric featuring alternating black and off-white threads. The reconstructed map accurately retained the sharp contrast and directional weave pattern, with clear stripe definition and no stitching artefacts.

• Colour Accuracy Analysis

To assess colour fidelity in a perceptually meaningful manner, the analysis focused on the dominant tones present in each albedo map rather than performing full pixel-wise comparisons, which are impractical for complex or non-repetitive fabric patterns. Instead, two visually representative colours were extracted from each sample to characterise the overall appearance, such as the combination of brown and dark brown in sample 1, or light and dark grey in sample 2.

Each dominant colour was converted to the CIE Lab colour space and compared with reference values obtained from professionally scanned textures. Colour differences were quantified using the CIEDE2000 (ΔE_00_) metric. As shown in Table 7, three out of four ΔE_00_ values fell below the perceptual threshold of 2.3, with the only exception being the darker tone of sample 1 [34]. Nonetheless, the average ΔE_00_ across both dominant colours remained within acceptable limits for each sample (2.25 for sample 1 and 1.86 for sample 2), indicating that overall colour accuracy is preserved. These results confirm that the proposed system can reliably reproduce perceptually representative colours, even for high-contrast or low-light tones.

• Texture Similarity Analysis

**Table 7:** Summary of dominant colours extracted from each albedo map and corresponding ΔE_00_ values compared with professionally scanned references. Colour swatches represent the reference and scanned tones for each identified hue.

To evaluate the preservation of structural details in the reconstructed albedo maps, a texture-based comparison was conducted using two complementary metrics: the Structural Similarity Index Measure (SSIM) and the Gray Level Co-occurrence Matrix (GLCM). SSIM quantifies global structural correspondence by comparing luminance, contrast, and local patterns between images, whereas GLCM statistically describes spatial intensity relationships, capturing local texture properties such as contrast, correlation, and homogeneity [35], [36].

As summarised in Table 8, Sample 1 achieved a high SSIM value (0.9848), confirming strong global agreement with the reference. In contrast, Sample 2 yielded a much lower SSIM (0.2474), even though the reconstructed and reference images appear visually consistent. This discrepancy stems from the striped, anisotropic pattern of Sample 2: SSIM is highly sensitive to minor geometric shifts or scale differences in repetitive textures, which cause phase mismatches despite perceptually similar appearances. To account for this limitation, GLCM-based homogeneity was additionally computed. The values remained consistently high for both samples (0.9697 and 0.9610), indicating that the local statistical texture distribution was accurately preserved regardless of alignment differences.

**Table 8:** Summary of texture similarity metrics (SSIM and GLCM homogeneity) between reconstructed and reference albedo maps.

Sample	SSIM	GLCM
#1	0.9848	0.9697
#2	0.2474	0.9611

It should be noted that the proposed evaluation is tolerant to slight misalignments or scale differences between the scanned and reference albedo maps, as the analysis focuses on perceptual and structural similarity rather than pixel-level correspondence. In practical applications, the textures can be rescaled or repositioned for visual alignment, and such minor geometric offsets do not affect the overall reproduction quality.

Overall, while SSIM performs well for globally aligned and isotropic textures, GLCM provides a more stable and perceptually relevant measure for complex or directionally patterned materials. These results confirm that the proposed system effectively preserves fine-scale texture characteristics even in the presence of small alignment variations.

### 7.2 Normal map assessment

The assessment of normal maps focused on capturing meaningful surface directionality that contributes to visual depth under projected lighting. Evaluation was based on both visual structure and directional distribution.

• Visual Inspection

**Fig. 18:**
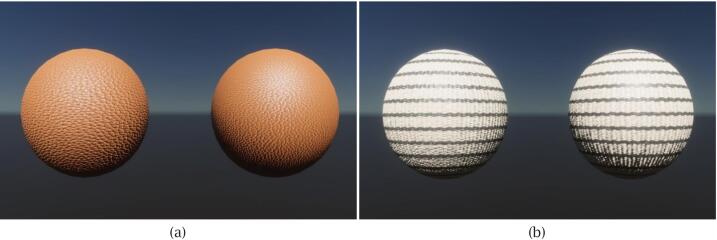
Visual comparison of normal maps applied to identical spherical surfaces in Unity for the two scanned fabrics. In each scene, the left sphere uses the reference normal map, and the right sphere uses the scanned normal map: (a) brown synthetic leather; (b) striped woven fabric.

To assess perceptual surface quality, the reference and scanned normal maps were applied to identical spherical surfaces rendered within a Unity HDRP (High Definition Render Pipeline) scene, allowing for enhanced visual fidelity. As shown in Fig. 18, the left sphere in each pair uses the reference normal map and the right sphere uses the scanned result. For both fabrics, the two renderings display comparable shading behaviour and relief features, indicating that the reconstructed normals capture the essential surface structure for P-SAR rendering.

• Directional Distribution Analysis

Although the scanned and reference normal maps may differ slightly in spatial extent and cropping scale—due to field-of-view limitations and alignment challenges—the high resolution of both images (4096 × 4096 pixels) ensures a statistically meaningful sample of surface normals. This allows for a valid comparison of their directional characteristics in a probabilistic manner, rather than relying on precise spatial correspondence.

**Fig. 19:**
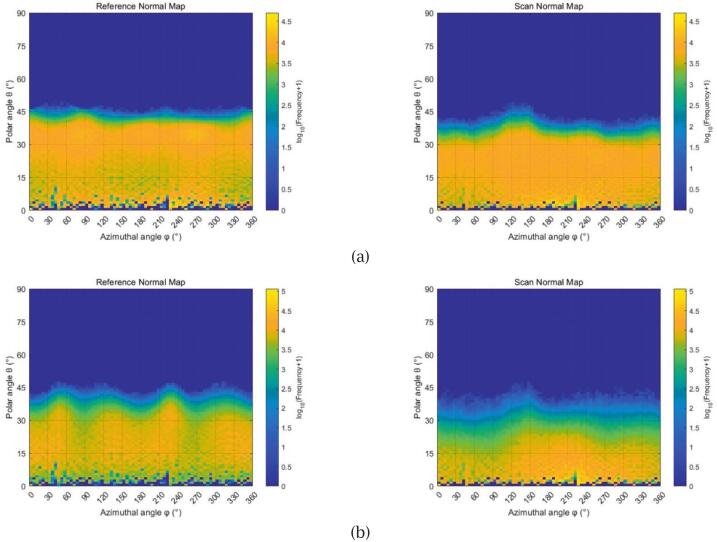
Normal orientation heatmaps (x: azimuthal angle φ in degrees, y: polar angle θ in degrees; colour: log-scaled frequency) for two fabric samples. Each row shows the reference (left) and scanned (right) normal maps: (a) sample 1, brown synthetic leather; (b) sample 2, striped woven fabric.

To analyse these characteristics, we generated spherical coordinate heatmaps that plot the azimuthal angle (φ) along the horizontal axis and the polar angle (θ) along the vertical axis. The colour encodes the logarithmic frequency of surface normals, providing an intuitive visualisation of orientation density across each texture. Fig. 19 compares the normal distributions from the proposed scanning system (right) against those from a professional reference (left) for two fabric samples.

In sample 1 (Fig. 19a), both the reference and scan maps exhibit dense distributions confined to low polar angles (approximately 0°–45°), resulting in a relatively flat contour along the azimuth. This reflects a planar surface with minimal geometric variation. The scan exhibits a slightly broader distribution, indicating minor angular dispersion, but overall retains the dominant directional pattern and surface flatness. In sample 2 (Fig. 19b), the reference map reveals a wavy contour along θ that repeats periodically across φ, indicative of structured anisotropy from the woven texture. The scanned map successfully captures this complex orientation trend, although with increased polar dispersion and slightly blurred pattern definition. These discrepancies highlight the challenge of reconstructing high-frequency geometry, yet the scan still conveys the essential directional features necessary for visually coherent rendering.

To quantitatively summarise these distributions, we computed two descriptive metrics: the angular range (min–max) and the mean direction for both polar and azimuthal angles. As shown in Table 9, the results from our scanner generally agree with those from the professional reference system, particularly for sample 1, where the differences are minimal. In contrast, sample 2 exhibits more noticeable deviations, especially in the mean polar angle (10.73° vs. 19.20°), which suggests a tendency to underestimate surface inclination. The azimuthal mean also shifts by over 15°, further indicating variation in directional consistency. These results suggest that while the scanner captures the general orientation distribution, directional accuracy may be compromised when dealing with more complex surface geometries.

**Table 9:** Summary of polar and azimuthal angle statistics for surface normals, including minimum, maximum, and mean values for each scanning sample.

Sample		Polar angle (θ)			Azimuthal angle (φ)		
		Min	Max	Mean	Min	Max	Mean
#1	Reference	0.32°	49.75°	21.97°	0.00°	359.78°	178.69°
	Scan	0.32°	49.64°	17.25°	0.00°	359.66°	183.57°
#2	Reference	0.32°	47.83°	19.20°	0.00°	359.73°	179.56°
	Scan	0.32°	47.48°	10.73°	0.00°	359.63°	196.61°

### 7.3 Workflow efficiency

To assess the practical viability of the proposed system, we recorded the time required for scanning and post-processing each fabric sample. The scanning time, measured from the initiation of the Arduino-controlled capture sequence to the completion of image acquisition, was approximately 15 s per sample and remained consistent due to full automation. Post-processing comprised three stages: image editing (cropping and white balance correction), reconstruction (importing into Substance 3D Designer to generate albedo and normal maps), and tiling (creating seamless textures). The total post-processing time averaged 253 s. Calibration time is excluded, as it mainly depends on image resolution and is not constrained by the scanning workflow. A detailed breakdown is provided in Table 10.

**Table 10:** Scanning and post-processing time for each scanning sample, with post-processing divided into three stages.

Sample	Scanning Time (s)	Post-processing Time (s)			Total Time (s)
#1	15	259			274
		Image Editing (s)	Reconstruction (s)	Tiling (s)	
		132	104	23	
#2	15	247			262
		Image Editing (s)	Reconstruction (s)	Tiling (s)	
		126	110	11	

Note that calibration time is excluded from this analysis, as it mainly depends on image resolution and is not constrained by the scanning workflow.

### 7.4 Limitations

The proposed scanning system is primarily suited for relatively flat, opaque materials. When applied to curved or bulky objects, self-shadowing and interreflections may occur, violating the assumptions of photometric stereo and leading to reconstruction inaccuracies. Although the use of a 3D LUT significantly improves colour reproduction, some hue groups—particularly those near the sensor’s spectral limits—may still show residual deviations. Moreover, larger directional errors were observed on anisotropic or high-frequency surfaces (e.g., sample 2), indicating that further improvements in normal estimation robustness under complex geometry are desirable.

The current validation mainly focuses on fabric materials, as A4-sized fabric samples were available and physically compatible with the present setup in terms of size, thickness, and mounting. Although only two representative fabrics are shown in this paper for clarity, additional tests on fabrics with different surface characteristics, such as woven textures, embossed leather-like structures, smooth synthetic textiles, and velvet-like diffusers, have been conducted, demonstrating the system’s adaptability within the fabric category. Future work will extend the validation to non-fabric opaque materials (e.g., wood, plastic, metal), for which accurate reconstructions are expected thanks to the cross-polarised illumination and colour-calibrated workflow. Conversely, translucent and transparent materials remain challenging under the current configuration; potential improvements include integrating upward-facing illumination beneath the sample holder to capture partial-transmission information.

## Ethics statements

This study does not involve human participants, animal subjects, or any ethical concerns requiring approval.

## Declaration of Generative AI and AI-assisted technologies in the writing process

During the preparation of this work, the authors used ChatGPT (OpenAI) in order to improve the readability, grammar, and clarity of the manuscript text. After using this tool, the authors reviewed and edited the content as needed and take full responsibility for the content of the published article.

## CRediT authorship contribution statement

**Lunan Wu:** Writing – original draft, Validation, Methodology, Investigation. **Federico Morosi:** Writing – review & editing, Supervision, Investigation, Conceptualization. **Giandomenico Caruso:** Writing – review & editing, Supervision, Investigation, Conceptualization.

## Declaration of competing interest

The authors declare that they have no known competing financial interests or personal relationships that could have appeared to influence the work reported in this paper.
